# New *Vibrio* species associated to molluscan microbiota: a review

**DOI:** 10.3389/fmicb.2013.00413

**Published:** 2014-01-02

**Authors:** Jesús L. Romalde, Ana L. Dieguez, Aide Lasa, Sabela Balboa

**Affiliations:** Departamento de Microbiología y Parasitología, CIBUS-Facultad de Biología, Universidad de Santiago de Compostela, Santiago de CompostelaSpain

**Keywords:** *Vibrionaceae*, genus *Vibrio*, molluscan microbiota, new species, pathogenicity, ecology

## Abstract

The genus *Vibrio* consists of more than 100 species grouped in 14 clades that are widely distributed in aquatic environments such as estuarine, coastal waters, and sediments. A large number of species of this genus are associated with marine organisms like fish, molluscs and crustaceans, in commensal or pathogenic relations. In the last decade, more than 50 new species have been described in the genus *Vibrio*, due to the introduction of new molecular techniques in bacterial taxonomy, such as multilocus sequence analysis or fluorescent amplified fragment length polymorphism. On the other hand, the increasing number of environmental studies has contributed to improve the knowledge about the family *Vibrionaceae* and its phylogeny. *Vibrio crassostreae, V. breoganii, V. celticus* are some of the new *Vibrio* species described as forming part of the molluscan microbiota. Some of them have been associated with mortalities of different molluscan species, seriously affecting their culture and causing high losses in hatcheries as well as in natural beds. For other species, ecological importance has been demonstrated being highly abundant in different marine habitats and geographical regions. The present work provides an updated overview of the recently characterized *Vibrio* species isolated from molluscs. In addition, their pathogenic potential and/or environmental importance is discussed.

## INTRODUCTION

Coastal and estuaries environments are growing areas of bivalve molluscs which become an important industry in many countries, due to the increasing importance of these animal as protein for human consumption. Due to their filter-feeding habit, bivalves accumulate a rich and diverse bacterial microbiota, composed of various species belonging to different genera like *Vibrio, Pseudomonas, Acinetobacter, Photobacterium, Moraxella, Aeromonas, Micrococcus*, and *Bacillus* (Murchelano and Brown, 1970; Kueh and Chan, 1985; Prieur et al., 1990).

Vibrios are Gram-negative curved rods that occur naturally in marine, estuarine, and freshwater systems worldwide. They occupy habitats ranging from the deep sea to shallow aquatic environments (Reen et al., 2006), being some species important for natural systems, including the carbon cycle and osmorregulation (Johnson, 2013), as free-living inhabitants in the water column or associated to particulate material. On the other hand, vibrios are also associated with a wide variety of poikilotherm and homoiotherm animals, including humans, for some of which are pathogens. Paillard et al. (2004) recognized that the emergence of vibrios as etiological agents of diseases is likely to increase over the coming years due to ocean warming.

The repeated episodes of mortality due to bacterial infections constitute one of the main problems in the culture of bivalve molluscs, since they reduce the production and cause high economical losses. Some members of the genus *Vibrio* have been described as the main aetiological agents of diseases affecting all life stages of molluscan shellfish (Liu et al., 2000; Allam et al., 2002; Waechter et al., 2002; Lee et al., 2003; Anguiano-Beltrán et al., 2004; Estes et al., 2004; Gay et al., 2004a, b; Paillard et al., 2004; Gómez-León et al., 2005; Prado et al., 2005; Labreuche et al., 2006a, b; Garnier et al., 2007, 2008).

The aim of the present work is to provide an overview on the diversity of *Vibrionaceae* associated with bivalve molluscs. Special emphasis is made on the species described in the last years in an attemp to clarify, not only their taxonomy, but also their pathogenic or ecological importance.

## VIBRIOS AS MICROBIOTA OF BIVALVE MOLLUSCS

In the literature, studies analyzing the diversity, distribution, and density of marine bacteria associated with bivalve molluscs are scarce. These studies date back to the 1960s and in general, results agree in the dominance of Gram negative over Gram positive bacteria in molluscs, as well as in the high abundance of bacteria belonging to the genus *Vibrio* (Colwell and Liston, 1960; Beenson and Johnson, 1967; Kueh and Chan, 1985).

From 1990s the diversity of *Vibrio* species associated with bivalves in different geographical areas has been the subject of various studies (Montilla et al., 1994; Hariharan et al., 1995; Arias et al., 1999; Pujalte et al., 1999; Maugeri et al., 2000; Caballo and Stabili, 2002; Castro et al., 2002; Guisande et al., 2004; Beaz-Hidalgo et al., 2008, 2010a; Lafisca et al., 2008). The main common conclusions obtained from these studies were that environmental parameters, such as variations in the water temperature and salinity, can influence the diversity of *Vibrio* spp. in the environment, as well as the physiological state of the bivalve and its susceptibility to bacterial infections (Arias et al., 1999; Pujalte et al., 1999; Maugeri et al., 2000; Paillard et al., 2004; Garnier et al., 2007).

In most studies, the predominating species associated with bivalves from different geographical locations (Spain, Canada, Italy, or Brazil), all from temperate climates, were either *V. splendidus, V. alginolyticus, V. harveyi*, or any combination of these species (Montilla et al., 1994; Arias et al., 1999; Pujalte et al., 1999). More recently, Beaz-Hidalgo et al. (2008) analyzed the diversity of *Vibrio* spp. in cultured Manila clams (*Venerupis philippinarum*) and carpet-shell clams (*Venerupis decusata*) by means of phenotypic and genotypic methods. The predominant species that accounted for 66.6% of the total identified strains were *Vibrio cyclitrophicus, V. splendidus*, and *V. alginolyticus*. Other species such as *V. fluvialis, V. vulnificus,* and *V. mimicus* have also been associated with molluscs (Maugeri et al., 2000; Caballo and Stabili, 2002). In those studies, the identification of *Vibrio* species was established using only phenotypic methods and, therefore, the real diversity present in bivalves could be underestimate. In fact, a high phenotypic variability was described within the *V. splendidus*-like and the *V. harveyi*-like groups which makes impossible to discriminate among several species (Thompson et al., 2005; Le Roux and Austin, 2006; Pascual et al., 2010).

As mentioned, most of the studies described the influence of environmental parameters (i.e., salinity and water temperature) on the diversity and alternance of *Vibrio* species (Kaspar and Tamplin, 1993; Motes et al., 1998; Arias et al., 1999; Pujalte et al., 1999). For instance, in bivalves from the Mediterranean Sea, *V. splendidus* has been found to be dominant during winter and spring and *V. harveyi* during the warmer months (Arias et al., 1999; Pujalte et al., 1999). Another example, in shellfish-growing areas of the US Northern Gulf Coast, the densities of *V. vulnificus* were high and almost constant at temperatures above 26°C and/or at salinity below 25 ppt, but decreased drastically below this temperature and/or above this salinity (Motes et al., 1998). The latter species together with *Vibrio parahaemolyticus* and *V. cholerae* are considered important human pathogens, producing important outbreaks after the consumption of contaminated shellfish (mainly oysters), and therefore have been the subject of many studies (Morris, 2003; Su and Liu, 2007; Jones and Oliver, 2009).

## NEW *Vibrio* SPECIES ASSOCIATED TO MOLLUSCS

The introduction of molecular techniques such as the fluorescent amplified fragment length polymorphism (FAFLP) and multilocus sequence analysis (MLSA) has allowed a more precise identification of *Vibrio* species which were previously masked under other taxa (Thompson et al., 2001, 2005; Beaz-Hidalgo et al., 2008, 2010a; Pascual et al., 2010).

In this sense, molecular studies have demonstrated the genetic diversity and the polyphyletic nature of *V. splendidus* (Thompson et al., 2001, 2005; Le Roux et al., 2002) and have enabled many new species to be described, such as *Vibrio kanaloae, V. pomeroyi, V. chagasii,* or *V. gallaecicus* (Thompson et al., 2003c; Beaz-Hidalgo et al., 2009b). Furthermore, phenotypically identified *V. harveyi* strains were re-classified as *V. campbellii* by FAFLP, DNA–DNA hybridization (DDH), and MLSA (Gomez-Gil et al., 2004a; Thompson et al., 2007). Pascual et al. (2010) investigated the usefulness of an MLSA approach with six housekeeping genes to discriminate six tightly related species with DDH values close to 70%, namely *V. harveyi, V. campbellii, V. rotiferianus, V. parahaemolyticus, V. alginolyticus*, and *V. natriegens*. They recognized the genes *tox*R (cholera toxin transcriptional activator) and *rpo*D (Sigma factor σ70) as the most reliable for species identification, and proposed a scheme for species definition on the basis of the similarities of the concatenated sequences of the most resolving genes.

In the last decade, more than 50 new species have been described in the genus *Vibrio*, many of them associated to marine environments and aquatic eukaryotic organisms. To mention some examples, among the new species described as free-living seawater bacteria are *V. agarivorans* (Macián et al., 2001b), *V. ruber* (Shieh et al., 2003), *V. aestivus* and *V. quintilis* (Lucena et al., 2012), *V. azureus* (Yoshizawa et al., 2009), or *V. sagamiensis* (Yoshizawa et al., 2010). Associated to different marine organisms have been described, among others, *V. caribbeanicus* from sponges (Hoffmann et al., 2012), *V. hemicentroti* from sea urchin (Kim et al., 2013), *V. corallilyticus, V. maritimus, V. shiloi, V. stylophorae*, and *V. variabilis* from corals (Kushmaro et al., 2001; Ben-Haim et al., 2003; Chimetto et al., 2011; Sheu et al., 2011), *V. rotiferianus* from rotifers (Gomez-Gil et al., 2003a), *V. comitans, V. gallicus, V. inusitatus, V. neonates, V. rarus*, and *V. superstes* from abalones (Hayashi et al., 2003; Sawabe et al., 2004a, b, 2007a), *V. atypicus, V. hispanicus, V. jasicida, V. owensii, V. pacinii, V. zhanjiangensis*, and *V. zhuhaiensis* from crustaceans (Gomez-Gil et al., 2003b, 2004b; Cano-Gómez et al., 2010; Wang et al., 2010; Jin et al., 2012, 2013; Yoshizawa et al., 2012), *V. hippocampi* from sea horses (Bálcazar et al., 2010), and *V. alfacsensis, V. sinaloensis*, and *V. tasmaniensis* from fish (Thompson et al., 2003d; Gomez-Gil et al., 2008, 2012).

**FIGURE 1:**
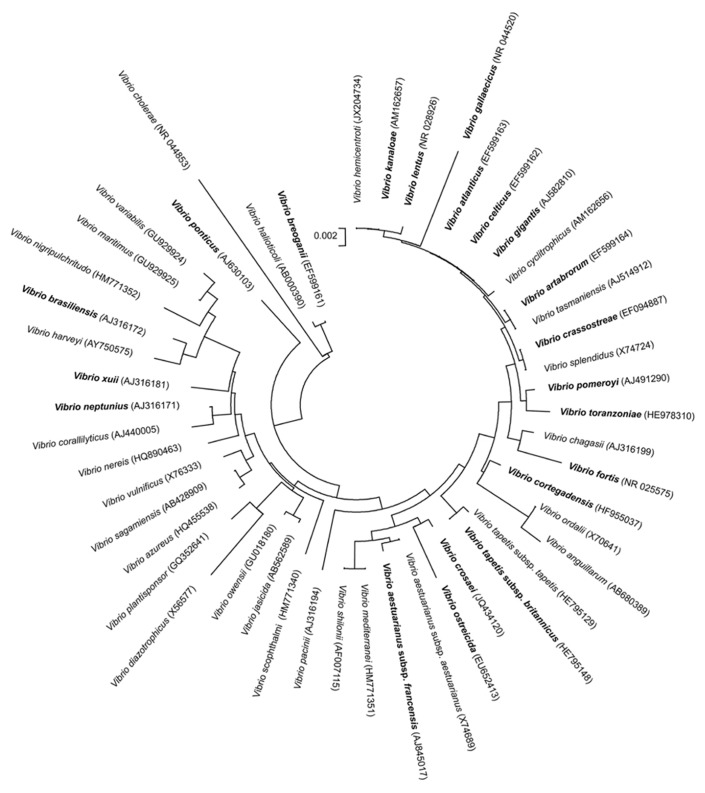
**Phylogenetic tree based on partial 16S rRNA gene sequences obtained by the neighbor joining method, including the *Vibrio* species associated with molluscs together with other representative species of the genus**. In bold are represented 19 new species and 2 new subspecies described since the turn of the century. GenBank sequence accession numbers of the correspondent type strains are given in parentheses. Bar, no. of substitutions per nucleotide position.

Regarding the vibrios described as associated with bivalve molluscs, and beside the well known species *V. alginolyticus, V. harveyi, V. mytili, V. parahaemolyticus, V. pectenicida,* or *V. vulnificus* (Pujalte et al., 1993; Lambert et al., 1998; Arias et al., 1999; Pujalte et al., 1999; Maugeri et al., 2000; Caballo and Stabili, 2002;Paillard et al., 2004; Beaz-Hidalgo et al., 2010a; Romalde et al., 2013), since the turn of the century 19 new species and 2 new subspecies have been described within the genus *Vibrio *(**Figure 1**). These new species and subspecies are listed below, in alphabetical order, including their key features, as well as their pathogenic potential and/or ecological relevance. It is noteworthythat about 50% of the new species belong to only one clade, Splendidus, comprising taxa phylogenetically closely related.

### *Vibrio* aestuarianus SUBSP. *Francensis* (Garnier et al., 2008)

The species *Vibrio aestuarianus* was described back by Tison and Seidler (1983) on the basis of a group of isolates obtained from oysters, clams and seawater in Oregon and Washington coasts (USA). Few years later, it was described as sharing close similarity with *Vibrio anguillarum*, and *V. pelagius* (Pillidge et al., 1987). This finding was confirmed 20 years later when Sawabe et al. (2007b) on the basis of MLSA of all known *Vibrio* species included *V. aestuarianus* within the Anguillarum clade of the genus. It has been described as a ubiquitous species in different geographic areas from the Baltic Sea (Eiler et al., 2006) to Hong Kong (Wang et al., 2006).

In the last years, it was associated with the syndrome known as “summer mortality” of the oyster (*Crassostrea gigas*) in the French coasts (Gay et al., 2004b; Garnier et al., 2007). The syndrome is thought to be multifactorial involving physiological and environmental factors as well as pathogens. Labreuche et al. (2006a, b) demonstrated the pathogenic potential of *V. aestuarianus* in experimental oyster challenges. The characterization of a group of isolates obtained from diseased oysters in France led to the description of a new subspecies, named *V. aestuarianus* subsp. *francensis*, on the basis of DDH values close to the boundary limit for species definition (70%) and several phenotypic differences with the American isolates.

### *Vibrio artabrorum* (Diéguez et al., 2011)

In a previous study (Beaz-Hidalgo et al., 2008), a collection of isolates obtained from Manila and carpet-shell clams and tentatively allocated to the genus *Vibrio* based on their phenotypic features were analyzed by FAFLP. One of the groups established, cluster 70, consisted of 8 isolates that could not be assigned to any of the known species of the genus *Vibrio*. Analysis of the 16S rRNA gene sequences allocated the isolates within the Splendidus clade forming a tight group. MLSA of five housekeeping genes, *atp*A (α-subunit of bacterial ATP synthase), *pyr*H [uridine monophosphate (UMP) kinase], *re*cA (RecA protein), *rpo*A (RNA polymerase α-chain), and *rpo*D, supported their inclusion in that clade forming a well differentiated group with respect to the rest of species, being its closest neighbors *V. pomeroyi* and *V. kanaloae*. DNA–DNA reassociation values confirmed its status of new species within the genus *Vibrio*. It is considered as an environmental species without pathogenic potential for clams.

### *Vibrio atlanticus* (Diéguez et al., 2011)

From the same study of Beaz-Hidalgo et al. (2008) another group of five strains, designated as cluster 5, was likely to be also a new *Vibrio* species, being further characterized using the same approach employed for the description of *V. artabrorum*. The phenotypical characterization, chemotaxonomy, MLSA, and DDH techniqeus confirmed the hypothesis that the clam isolates constituted a new species, related with *V. tasmaniensis, V. kanaloae*, and *V. cyclitrophicus* within the Splendidus clade. As *V. artabrorum*, and since until now no pathogenic activity can be proved for *V. atlanticus*, it seems to be part of the normal environmental and clam microbiota.

### *Vibrio brasiliensis* (Thompson et al., 2003a)

Six isolates obtained from lion’s paw scallop (*Nodipecten nodosus*) larvae were identified as a tight group during a wide study on vibrios by FAFLP (Thompson et al., 2001). Further characterization of those isolates on the basis of phenotypic features, 16S rRNA gene sequencing, G + C content and DDH, allowed the description of the new species *V. brasiliensis* within the Orientalis clade. Its pathogenic potential was demonstrated in experimental challenges using rainbow trout (*Oncorhynchus mykiss*) and *Artemia nauplii* as animal models (Austin et al., 2005). The extracellular products (ECP) of the strain tested were also harmful to the animals.

### *Vibrio breoganii* (Beaz-Hidalgo et al., 2009a)

A group of seven non-motile, facultative anaerobic alginolytic marine bacteria isolated from cultured Manila and carpet-shell clams in Galicia (NW Spain) were characterized employing a polyphasic approach, including the sequence analysis of the 16S rRNA gene and three housekeeping (atpA, recA, and rpoA) genes, FAFLP fingerprinting, G + C content, DDH, and phenotypic features. Phylogenetic analysis based on 16s rRNA gene sequences showed that the isolates were closely related to the species *V. comitans, V. rarus,* and *V. inusitatus*, with sequence similarities of approximately 99%. However, phylogenies based on the sequences of the housekeeping genes grouped the isolates together and allocated them within the Halioticoli clade, although they can be differentiated from the other species in the clade by their FAFLP profiles. DDH experiments confirmed that they represented a new *Vibrio* species, that was named *V. breoganii*.

Some years later, this species was consistently detected in a meta-analysis of three separated studies aimed to identify the ecological population structure of *Vibrionaceae* in the Plum Island Sound Estuary (Ipswich, MA, USA), mainly associated to large particles and zooplankton (Preheim et al., 2011). Interestingly, population of *V. breoganii* showed essentially identical results with respect to genetic breadth in all studies, regardless the season or the sampling method. This species constitutes a good example of how genotypic clusters established by MLSA can serve as a reasonable initial definition of cohesive unit from an ecological point of view, as well as of the ubiquity of *Vibrio* species in the marine environment.

In 2012, a strain of this species was included in one of the first studies examining the complete methylation pattern of a bacterial genome (Murray et al., 2012). The knowledge of the methylome could be of great interest due to the recognized importance of methylation for understanding fundamental microbiological processes, microbe adaptability, and disease pathogenicity. 

### *Vibrio celticus* (Beaz-Hidalgo et al., 2010b)

A group of four motile facultative anaerobic marine isolates obtained from cultured pullet carpet-shell (*Venerupis pullastra*) and Manila clams during 2004 and 2005 in Galicia (NW Spain) were studied using a polyphasic approach. It was found that they formed a tight phylogenetic group based on sequences of the 16S rRNA gene and four housekeeping (*atp*A, *rec*A, *rpo*A, and *rpo*D) genes, indicating that the four isolates represented a novel species in the Splendidus clade of the genus *Vibrio*, for which the name *V. celticus* was proposed. In addition, the strains showed potential pathogenic activity for adult clams in virulence assays. 

Recently, a study on the diversity of *Vibrio* spp. in the Eastern English Channel by means of sequencing of the housekeeping gene* pyr*H (Tall et al., 2013), revealed that *V. celticus *was the predominant species among other 20 *Vibrio* species isolated at ambient environmental temperature.

### *Vibrio cortegadensis* (Lasa et al., 2013b)

It was described as a results of the polyphasic characterization of a group of four marine strains isolated from carpet-shell and Manila clams in Galicia (NW Spain). The study of the phenotypic characteristics, the analysis of chemotaxonomic features, the sequencing of the 16S rRNA and five housekeeping (*atp*A, *pyr*H, *rec*A, *rpo*A, and *rpo*D) genes, as well as DDH, allowed the identification of the isolates within the genus *Vibrio*, being their closest neighbors *V. tapetis, V. pomeroyi,* and *V. crassostreae* (97.9%). The phylogenetic analysis of the five concatenated genes indicated the allocation of these strains in between the Splendidus and Anguillarum clades.

### *Vibrio crassostreae* (Faury et al., 2004)

Described in 2004 on the basis of five strains obtained from oyster haemolimph, and originally identified as *V. splendidus*-like isolates. The authors employed a polyphasic approach including besides biochemical tests, fatty-acid methyl ester (FAME) analysis, 16S rRNA, and *gyr*B (DNA gyrase subunit B) genes sequencing, FAFLP fingerprinting, and DDH. Although all the genetic studies supported that the five strains constituted a novel *Vibrio* species within the Splendidus clade, their differentiation of the closest relatives was not possible on the basis of 17 phenotypic characters. However, the presence of fatty acids 16:0 iso and 14:0 iso allowed the differentiation of the new species from other *V. splendidus*-like species. It was described as a species with pathogenic potential for the oyster *C. gigas* (Gay et al., 2004a).

### *Vibrio crosaei* (González-Castillo et al., 2014)

The description of this new *Vibrio* species was based on the study and characterization of one isolate obtained from cultured oyster (*C. gigas*) in Sonora (Mexico). The phenotypic characteristics and the 16S rRNA gene sequence of the isolate clearly placed it within the genus *Vibrio*, with *V. orientalis* and *V. rotiferianus* as closest relatives. Curiously, these both species belong to different clades, as proposed by Sawabe et al. (2007b), the Orientalis clade and the Harveyi clade, respectively. MLSA technique clarified the definitive allocation of the isolate within the Orientalis clade, and the DNA relatedness measures by DDH experiments confirmed that it constituted a new *Vibrio* species. The proposed name, *Vibrio crosaei*, was chosen to honor Prof. Dr. Jorge Crosa, microbiologist and specialist in vibrios.

### *Vibrio fortis* (Thompson et al., 2003b)

The species was defined on the basis of 10 isolates obtained between 1994 and 1999 from different hosts, including healthy and diseased lion’s paw scallop larvae, diseased *C. gigas* larvae, shrimp (*Litopenaeus vannamei*) larvae, Atlantic salmon (*Salmo salar*), as well as sea water. The geographical origins included different North and South American Countries, Tasmania, and UK. Genotypic analysis such as 16S rRNA gene sequencing and DDH confirmed the deliniation of this new species, differentiating it from the closets neighbors *V. pelagius* or *V. mytili*. Austin et al. (2005) confirmed the pathogenic potential of the species using rainbow trout and *A. nauplii* as animal models.

*Vibrio fortis* was further isolated from spotted rose snapper (*Lutjanus guttatus*) in Mexico (Gomez-Gil et al., 2007) and from crown-of-thorns starfish (*Acanthasther planci*) in Australia and Guam (Rivera-Posada et al., 2011). It was also identified as one of the predominant *Vibrio* species in the Cariaco Basin, Venezuela (García-Amado et al., 2011).

The extracellular polymeric substances from this biofilm forming *Vibrio* species were characterized (Kavita et al., 2013), showing potential for industrial applications.

### *Vibrio gallaecicus* (Beaz-Hidalgo et al., 2009b)

This species within the Splendidus clade was described on the basis of the characterization of three strains isolated from Manila clams in Galicia (NW Spain). Phylogenetic analysis of the 16S rRNA gene and four housekeeping (*atp*A, *pyr*H, *rec*A, and *rpo*A) genes, indicated that these strains were closely related to the Splendidus clade, being its closest relatives *V. splendidus, V. gigantis,* and *V. pomeroyi*. The FAFLP fingerprints and DDH values supported the MLSA results. It is considered as an environmental species without proved pathogenic potential.

### *Vibrio gigantis* (Le Roux et al., 2005)

The polyphasic characterization of four isolates obtained from *C.* *gigas* haemolymph allowed the description of this new *Vibrio* species within the Splendidus clade. Although 16S rRNA gene sequence analysis did not permit a clear differentiation of *V. gigantis* from other phenotypically related species, other techniques including FAFLP, DDH, and sequencing of four housekeeping [*gyr*B, *rct*B (replication origin-binding protein), *rpo*D, and *tox*R] genes demonstrated that the isolates formed a tight genomic group, clearly differentiated from the neighboring species. The authors suggested that, as other *Vibrio* species present in the shellfish haemolymph, *V. gigantis* may play a role in the health of the host.

### *Vibrio kanaloae* (Thompson et al., 2003a)

It was described on the basis of five isolates with different origins, including diseased oyster (*Ostrea edulis*) larvae from France, shrimp (*Penaeus chinensis*) from China and sea water from Hawaii (USA). Therefore, it has been described as an ubiquitous species in the aquatic environment. The five strains were originally detected in a wide FAFLP study (Thompson et al., 2001) as a separate cluster, showing a pattern clearly different from other *Vibrio* species, with which share the main phenotypic traits of the genus. Further DDH experiments confirmed that they were in fact a new species within the Splendidus clade.

Later studies on the virulence of other related strains were performed on the basis of experimental infections of *C. gigas*. After injection of strains, bacteria were localized at the periphery of the muscle and induced extensive lesions of the translucent part of the adductor muscle. Unfortunately, although using a polyphasic approach these strains were confirmed to be *V. splendidus*-related, no clear discrimination between *V. kanaloae* and *V. pomeroyi* was possible with the techniques employed. (Gay et al., 2004b). Austin et al. (2005) confirmed its pathogenic potential for aquatic animals including fish and crustaceans.

### *Vibrio lentus* (Macián et al., 2001a)

The study of 12 marine bacteria by means of cultural and physiological characterization, ribotyping, G+C content, DDH, and phylogenetic analysis on the 16S and 23S rRNA genes allowed the description of this new *Vibrio* species in 2001. All the strains had been isolated from Mediterranean oysters in Spain, and were phenotypically similar to *V. splendidus*. The name *V. lentus* was proposed since the strains showed a slow growth on Marine Agar. Thus, colonies of some of the isolates were not larger than 0.2 mm diameter after 3 days of incubation. Some years later *V. lentus* was isolated from diseased wild octopus (*Octopus vulgaris*) and from turbot (*Scophthalmus maximus*) also in Spain (Farto et al., 2003; Montes et al., 2003, 2006). In the case of octopus, experimental infections by bath challenge demonstrated that *V. lentus* was able to reproduce the skin lesions, colonize the internal organs, and induce mortality in healthy octopuses (Farto et al., 2003).

The presence of a lethal extracellular 39-kDa protease, similar to that of *Vibrio pelagius*, was detected in 15% of the ECP assayed belonging to strains of the *Vibrio splendidus*–*V. lentus* related group by Farto et al. (2006), which suggested their potential risk for the health of reared aquatic organisms.

### *Vibrio neptunius* (Thompson et al., 2003c)

Described during a polyphasic study of 21 isolates with diverse origins, like healthy and diseased lion’s paw scallop larvae, rotifers, and turbot larvae. The results clearly indicated that this group od strains constituted a new species within the Corallilyticus clade of the genus. *Vibrio neptunius* was further identified as aetiological agent of a mortality episode of oyster (*O. edulis*) larvae occurred in a Galician hatchery (Prado et al., 2005). Pathogenicity was confirmed in experimental tests where it shown to cause high mortalities (ranging from 98.5 to 100%) in 72 to 96 h after inoculation of larval cultures. The work of Prado and co-workers constituted the first description of *V. neptunius* as a molluscan pathogen. Later studies with New Zealand Greenshell mussel (*Perna canaliculus*) larvae confirmed the pathogenic potential for other molluscan species (Kesarcodi-Watson et al., 2009a,b).

*Vibrio neptunius* was also found in environmental studies as a predominant bacteria in the anoxic zone (García-Amado et al., 2011). In addition, during a study searching for novel antimicrobials in marine *Vibrionaceae* (Wietz et al., 2010), *V. neptunius* has been identified as a potential resource of antibacterial compounds with future applicability.

### *Vibrio ostreicida* (Prado et al., 2014)

The species description relies on three strains isolated from a flat oyster (*O. edulis*) hatchery in Spain after episodes of high mortality (Prado et al., 2005). Pathogenicity was confirmed in experimental tests where the strains were able to cause high larval mortalities. The results of the phenotypic and genotipic analysis revealed that this group of strains constituted a new *Vibrio* species, closely related to *V. pectenicida*.

### *Vibrio pomeroyi* (Thompson et al., 2003a)

As in the case of *V. kanaloae, V. pomeroyi* was originally detected as a group of four isolates showing a characteristic FFAFLP pattern during a study on the genomic diversity amongst *Vibrio* isolates from different sources (Thompson et al., 2001). Two strains had been isolated from healthy bivalve larvae (*N. nodosus*) in Brasil and two from turbot in Spain. They were confirmed as a new *Vibrio* species within the Splendidus clade by means of DDH, phenotypic characterization, and FAME analysis. The studies of Gay et al. (2004a) and Austin et al. (2005) mentioned before demonstrated either non- or low virulence of *V. pomeroyi* in animal models. 

### *Vibrio ponticus* (Macián et al., 2004)

It has been described to accommodate four marine bacteria isolated from mussels, fish, and seawater at the Mediterranean coast of Spain. Phylogenetic analysis locate these strains in the vicinity of the Fluvialis–Furnissii clade, sharing with these species similarities slightly higher tan 97% in their 16S rRNA gene sequences. Since one of the isolates were isolated after direct plating of a kidney sample from a diseased gilthead seabream (*Sparus aurata*), the pathogenic potential of the species cannot be discarded.

### *Vibrio tapetis* subsp. *britannicus* (Balboa and Romalde, 2013)

*Vibrio tapetis*, described by Borrego et al. (1996), is the causative Vibriotapetis, describedby Borrego et al. (1996), isthecausativeagent of an epizootic infection described in adult clams called Brown Ring Disease (BRD) constituting a major limiting factor for the culture of Manila clams. This pathogen was considered for years as a highly homogeneous taxon on the basis of its phenotypical features, but the isolation of new strains from different hosts revealed some variability both at serological and genetic level, allowing the description of three major groups related to the host origin of the isolates (Castro et al., 1997; Romalde et al., 2002; Rodríguez et al., 2006). Balboa and Romalde (2013) performed for the first time a phylogenetic study for this pathogen, where *V. tapetis* strains appeared clearly separated in two main robust clusters, one containing the isolates from the British Isles and other one containing the isolates from all other geographic origins. The two clusters, that showed values of DDH between 65.05 and 79.8%, were easily distinguishable for their capacity to produce acid from mannitol and arabinose and for the use of citrate. On the basis of these results, not only an emended description was provided for *V. tapetis*, but also the new subspecies *V. tapetis *subsp. *britannicus* was proposed.

### *Vibrio toranzoniae* (Lasa et al., 2013a)

It was recently described in a polyphasic study of four strains isolated from cultured carpet-shell and Manila clams in the Northwest of Spain. The techniques utilized included phylogenetic analysis based on sequences of 16S rRNA and MLSA of five housekeeping genes (*atp*A, *rec*A, *pyr*H, *rpo*A, and *rpo*D), DDH, FAME analysis and more than 100 phenotypic traits. All the closest relatives were *Vibrio* species included in the Splendidus clade, such as *V. kanaloae, V. artabrorum, V. gigantis,* or *V. celticus*, from which it can be easily differentiated by several phenotypic characteristics. Current studies of some Chilean *Vibrio* strains isolated from fish seem to indicate that the geographical and host distribution of this species could be wider than expected.

### *Vibrio xuii* (Thompson et al., 2003c)

Three isolates obtained from bivalve and shrimp systems were identified as a tight group during a wide study on vibrios by FAFLP (Thompson et al., 2001). Further characterization of those isolates on the basis of phenotypic features, 16S rRNA gene sequencing, G + C content, and DDH, allowed the description of the new species *V. xuii* within the Nereis clade. Considered as an environmental species, *V. xuii* demonstrated either non- or low virulence in the animal models (Austin et al., 2005).

## MOLLUSC AND OTHER *Vibrionaceae*

Other* Vibrionaceae *described in the last years also associated to molluscan shellfish are representatives of the genera *Aliivibrio* and* Photobacterium*.

The genus *Aliivibrio* was established by Urbanczyk et al. (2007) to accommodate the species *V. fischeri, V. logei, V. salmonicida*, and *V. wodanis*, on the basis of a study based on 16S rRNA gene sequencing and MLSA which results indicated that the four species represented a lineage within the *Vibrionaceae* distinct from other genera. Therefore, the authors proposed the reclassification of the species as *Aliivibrio fischeri* (the type species), *A. logei, A. salmonicida* and *A. wodanis*, respectively. The genus includes symbiotic (*A. fischeri*) and pathogenic (*A. salmonicida*) species for marine organisms (Urbanczyk et al., 2007).

*Photobacterium* was one of the oldest established genus in the family* Vibrionaceae. *The type species is *Photobacterium phosphoreum*, which had been described by Cohn in 1878 as “*Micrococcus phosphoreus*” (Gomez-Gil et al., 2011). At the time of writing, the genus *Photobacterium* contained 23 species (). Although most species have no described pathogenic activity and are common inhabitants of marine environment, some species, i.e., both subspecies of *Photobacterium damselae*, are pathogenic for aquatic animals, mainly for fish.

### *Aliivibrio finisterrensis* (Beaz-Hidalgo et al., 2010c)

This species was described after the phenotypic and genotypic characterization of four strains isolated from cultured Manila clam in the north-western coast of Spain. Phylogenetic analyses based on the 16S rRNA gene sequences indicated that these bacteria were closely related to *A. wodanis, A. salmonicida, A. fischeri*, and *A. logei* with sequence similarities between 98.1 and 96.0%. Phylogenetic analysis based on MLSA of four housekeeping genes and FAFLP experiments clearly showed that these novel isolates form a tight genomic group different from any currently known *Aliivibrio* species.

### *Photobacterium swingsii* (Gomez-Gil et al., 2011)

The characterization of six Gram-negative coccobacilli, isolated from Pacific oysters (*C. gigas*) from Mexico and haemolymph of spider crabs (*Maja brachydactyla*) from Spain, allowed the description of this species within one of the oldest established genera in the family *Vibrionaceae*. Repetitive palindromic PCR (REP-PCR) analysis revealed a high degree of genomic homogeneity among the isolates. Several phenotypic traits differentiated the isolates from the type strains of species of the genus *Photobacterium*, including its closest relatives *P. aplysiae* and *P. frigidiphilum*.

## FUTURE PERSPECTIVES

Although classification of bacteria into a natural system has been hampered by the lack of a generally applicable species concept, the introduction of MLSA has provided much higher resolution for microbial identification and taxonomy (Gevers et al., 2005). The groups or species defined by means of MLSA are of particular interest for microbial ecology, since some theories predict that they correspond to ecologically cohesive populations (Fraser et al., 2009; Preheim et al., 2011). Some examples have been mentioned in this review, such as *V. breoganii* or *V. celticus* which, some years after their description as species, have been identified in ecological studies in different geographical areas as predominant populations. It is likely expected that in the near future more efforts will be made to identify ecological populations using these or other approaches, including single-cell amplification of multilocus genes or single-cell genomics (Stepanauskas and Sieracki, 2007; Rodrigue et al., 2009). As indicated by Preheim et al. (2011), to establish reproducible associations between bacterial species and environmental categories may be helpful to predict their occurrence and to get a deeper knowledge on the ecological factors driving their evolution.

It has been indicated that a phylogenetic hypothesis based on complete genomes is desired for *Vibrionaceae* (Dikow and Smith, 2013), and the new pyrosequencing and bioinformatic tools available would be very helpful to obtain such goal. Comparative genome analyses have already revealed a variety of genomic events, including mutations, chromosomal rearrangements, loss of genes by decay or deletion, and gene acquisitions through duplication or horizontal transfer (e.g., in the acquisition of bacteriophages, pathogenicity islands, and super-integrons), that are probably important driving forces in the evolution and speciation of vibrios (Hazen et al., 2010; Morrison et al., 2012; Daccord et al., 2013; Dikow and Smith, 2013).

On the other hand, a better knowledge of the in situ or real-time function of vibrios is needed, both in the environment or within the microbiota of aquatic animals (Frias-Lopez et al., 2008; Jones et al., 2008). Metatranscriptomics would be a valuable method, not only to reveal “near instantaneous” responses to environmental changes, but also to determine the real role of vibrios in different habitats or hosts.

## CONCLUDING REMARKS

The present study overviewed the diversity of *Vibrio* species associated with bivalve molluscs. Ongoing studies on the disease and pathogenicity of bivalves primarily relies on the use of phenotypic and molecular methods for an exact species identification. It remains to be investigated to what extent some of the recently discovered species are commensal, opportunistic or pathogenic organisms. Knowledge of the infection mechanisms used by classical and emerging *Vibrio* spp. to develop disease in bivalve molluscs will help to establish adequate preventive measures to control the transmission of these pathogens in hatcheries and in coastal growing areas.

Finally, and as pointed out by Grimes et al. (2009), future information on completed genomes, metagenomics, and metatranscriptomics will increase the understanding on the biology and ecology of vibrios, providing new insights and solutions to problems with disease, nutrient cycling in the ocean, and opportunities in marine biotechnology.

## Conflict of Interest Statement

The authors declare that the research was conducted in the absence of any commercial or financial relationships that could be construed as a potential conflict of interest.
